# Dynamic alterations of amplitude of low-frequency fluctuations in patients with chronic neck pain

**DOI:** 10.1093/psyrad/kkab011

**Published:** 2021-08-27

**Authors:** Jiabao Zhang, Tao Xu, Linjia Wang, Dan Chen, Lisha Gong, Huafu Chen, Jiali Yu, Ling Zhao, Qing Gao

**Affiliations:** School of Mathematical Sciences, University of Electronic Science and Technology of China, Chengdu, 611731, China; Acupuncture and Tuina School, Chengdu University of Traditional Chinese Medicine, Chengdu 610075, China; Acupuncture and Tuina School, Chengdu University of Traditional Chinese Medicine, Chengdu 610075, China; Acupuncture and Tuina School, Chengdu University of Traditional Chinese Medicine, Chengdu 610075, China; School of Mathematical Sciences, University of Electronic Science and Technology of China, Chengdu, 611731, China; Department of Radiology, First Affiliated Hospital to Army Medical University, Chongqing, 400038, China; High-Field Magnetic Resonance Brain Imaging Key Laboratory of Sichuan Province, School of Life Science and Technology, University of Electronic Science and Technology of China, Chengdu, 610054, China; School of Mathematical Sciences, University of Electronic Science and Technology of China, Chengdu, 611731, China; Acupuncture and Tuina School, Chengdu University of Traditional Chinese Medicine, Chengdu 610075, China; School of Mathematical Sciences, University of Electronic Science and Technology of China, Chengdu, 611731, China

**Keywords:** chronic neck pain, resting-state functional magnetic resonance imaging, local brain activity, dynamic amplitude of low-frequency fluctuations, dynamic variability

## Abstract

**Background:**

The pathogenesis of neck pain in the brain, which is the fourth most common cause of disability, remains unclear. Furthermore, little is known about the characteristics of dynamic local functional brain activity in cervical pain.

**Objective:**

The present study aimed to investigate the changes of local brain activity caused by chronic neck pain and the factors leading to neck pain.

**Methods:**

Using the amplitude of low-frequency fluctuations (ALFF) method combined with sliding window approach, we compared local brain activity that was measured by the functional magnetic resonance imaging (fMRI) of 107 patients with chronic neck pain (CNP) with that of 57 healthy control participants. Five pathogenic factors were selected for correlation analysis.

**Results:**

The group comparison results of dynamic amplitude of low-frequency fluctuation (dALFF) variability showed that patients with CNP exhibited decreased dALFF variability in the left inferior temporal gyrus, the middle temporal gyrus, the angular gyrus, the inferior parietal marginal angular gyrus, and the middle occipital gyrus. The abnormal dALFF variability of the left inferior temporal gyrus was negatively correlated with the average daily working hours of patients with neck pain.

**Conclusions:**

The findings indicated that the brain regions of patients with CNP responsible for audition, vision, memory, and emotion were subjected to temporal variability of abnormal regional brain activity. Moreover, the dALFF variability in the left inferior temporal gyrus might be a risk factor for neck pain.

This study revealed the brain dysfunction of patients with CNP from the perspective of dynamic local brain activity, and highlighted the important role of dALFF variability in understanding the neural mechanism of CNP.

## Introduction

According to the 2010 global burden of disease study, neck pain is the fourth leading cause of disability after back pain, depression, and joint pain (Hogg-Johnson *et al*., 2009; Hoy *et al*., 2010). The occurrence and development of neck pain may be related to genetic, psychology (anxiety, depression, somatization symptoms, etc.), sleep, smoking, and sedentary lifestyle (Elbinoune *et al*., 2016; Park *et al*., 2016). Chronic neck pain (CNP) refers to pain that lasts for more than 3 months and is aggravated after some activities, and likely accompanied by dizziness, anxiety, and insomnia at the same time. Evidence has shown that cervical spondylosis could lead to structural damage to the spinal cord and long-term nerve damage, including neck pain and motor weakness (Woodworth *et al*., 2019). Although CNP has affected 10–24% of the global population (Hoy *et al*., 2010), its pain is persistent, chronic, and less sharp than other acute pains whereby it was assumed to make little difference to brain structure or function. As a result, fewer studies on CNP have examined neural alteration (Spisak *et al*., 2020). Previous studies have proposed that CNP etiology may be attributed to abnormal muscle control, but there is inadequate knowledge about the complex pathogenesis so far (Falla *et al*., 2003; Falla *et al*., 2004).

There have been studies trying to find the pathogenesis of CNP in the brain (Lutz *et al*., 2008; Cagnie *et al*., 2014; Kim *et al*., 2014; Lieberman *et al*., 2014; De Pauw *et al*., 2019; Woodworth *et al*., 2019), i.e. the changes in brain structure or function in patients. Patients with advanced neck pain have been found to have aberration of cortical thinning and atrophy, neurological deterioration, and pain symptoms in specific brain regions related to sensory motor and pain management (Woodworth *et al*., 2019). However, DePauw *et al*. found that in chronic idiopathic neck pain (CINP), the left precuneus cortex and the left superior parietal gyrus were thicker and the volume of the left parietal gyrus was larger in patients than that in normal participants (De Pauw *et al*., 2019). Overall, studies have shown that gray matter volume, cortical thickness, and white matter structure in the brain regions involved in pain and cognition change to varying degrees in patients with CNP (Lutz *et al*., 2008; Cagnie *et al*., 2014; Kim *et al*., 2014; Lieberman *et al*., 2014).

Resting-state brain activity reflects aspects of the intrinsic property of brain fluctuation organization (Raichle and Snyder, 2007). The research on brain function of patients with CNP focused on static methods, such as regional homogeneity, functional connectivity, amplitude of low-frequency fluctuations (ALFF), and so on. For example, regional brain activity studies (Chen *et al*., 2018; Ihara *et al*., 2019) have shown that CNP exhibits abnormal regional homogeneity values in the bilateral medial superior frontal gyrus and right auxiliary motor areas, as well as abnormal functional connectivity values in the left middle frontal gyrus and the bilateral amygdala. Most of these studies relied on an implicit assumption that brain activity remains stationary during functional magnetic resonance imaging (fMRI) scanning. However, recent studies proposed that brain activity is dynamic over time (Hutchison *et al*., 2013; Allen *et al*., 2014). One study proposed that the ALFF is an effective approach for measuring local brain activity (Zang *et al*., 2007). Additionally, brain activity is dynamic and the dALFF method has been proposed to measure the variance of ALFF over time. The dALFF provides a new avenue to depict time-varying local brain activity (Rong *et al*., 2018) and has been applied in many fields. However, knowledge about whether patients with CNP exhibit abnormal dynamic local brain activity remains vague. Recognizing these abnormalities would improve our understanding about the neuropathological mechanisms of CNP, and thereby promote the development of effective treatment for CNP.

In this study, the ALFF approach and sliding window method were selected to explore the dynamic local brain activity in patients with CNP. It is hypothesized that these patients would exhibit a different dALFF pattern compared with healthy control (HC) groups, which may contribute to exploring the pathogenesis of neck pain in brain.

## Materials and Methods

### Participants

A total of 107 patients with CNP were recruited from the acupuncture and orthopedic clinics of the hospital of Chengdu University of Traditional Chinese Medicine. A group of 57 HCs also participated in the study.

All participants were right-handed and were aged between 18 and 75 years. The patients met the diagnostic criteria for CNP, which is defined in the Guidelines for Diagnosis, Treatment and Rehabilitation of Cervical Spondylosis compiled by the Professional Committee of China Rehabilitation Medical Association in 2010, and the course of disease was more than 3 months.

The main exclusion criteria included: (i) complications with other serious organic diseases, including malignant tumor, tuberculosis, fracture, and osteomyelitis; (ii) complications with serious primary diseases, such as cardiovascular, cerebrovascular, liver, kidney, and the hematopoietic system; (iii) mental disorders that could be matched with the mental questionnaire for the diagnosis of mental diseases; (iv) a bleeding tendency, allergic constitution, and skin diseases; (v) pregnant and lactating women, and those who had fertility experiences in the past 6 months; and (vi) people who were participating in other clinical trials.

The VAS and the short form of McGill pain questionnaire (SF-MPQ) were used to evaluate the degree of neck pain and neck disability index (NDI). The 12-question short form (SF-12) was used to evaluate the impact of neck pain and its intensity on daily life. Patients with CNP and HC were matched for age (Table 1). All participants were provided with information about the research procedure and written informed consent before the experiment. This study was approved by the supervision of the Sichuan Regional Ethics Review Committee on TCM (ethical approval number 2018KL-056) and was registered in the Chinese Clinical Trial Registry (registration number ChiCTR1800017718).

**Table 1:** Demographics and clinical data.

Variable	Patients (*n* = 106)	HCs (*n* = 57)	*P* value
Age (years)	46.23 ± 13.78	42.35 ± 13.00	0.1
Sex (male/female)	28/78	10/47	—
Duration of illness (months)	84.96 ± 74.00	—	—
Daily average desk time (hours)	4.84 ± 3.20	—	—
Seizure frequency per month (days)	6.18 ± 0.97	—	—
Daily average digital products time (hours)	3.99 ± 2.79	—	—
Seizure frequency per day (hours)	8.81 ± 6.66	—	—

The *P* value was obtained by a two-sample *t-*test (two-tailed).

### MRI data acquisition

MRI images were acquired on a GE 750 3.0T MRI system in the MRI center of the Affiliated Hospital of Chengdu University of Traditional Chinese Medicine. During the scan, the participants were instructed to lie down with their eyes closed, muscles relaxed, and not to fall asleep or substantially move their head. Padded foam pads were used to restrict head motion, and earplugs used to attenuate scanner noise. After the structure image was positioned in the conventional three planes, the axial scanning of the structure image was performed using a T1 weighted fast disturbing phase gradient echo sequence. The scanning parameters were: TR/TE = 2530 ms/3.4 ms, field of view = 240 × 240 mm, matrix size = 512 × 512, turning angle 12° and layer thickness of 1 mm. The gradient echo sequence of single shot planar echo was used for functional imaging and its scanning parameters were: TR/TE = 2000 ms/30 ms, matrix = 64 × 64, field of view = 240 × 240 mm, turning angle 90°, slice thickness = 5 mm, continuous scanning without septum and voxel size = 3.75 × 3.75 × 5 mm. The scanning included the whole brain, cerebellum, and brainstem.

### Data preprocessing

The original DICOM format images were transmitted to the workstation and the integrity of the original data was checked first. The quality of the original data was then checked using MRIcron software. After eliminating any images with large artifacts and other obvious image quality problems, such as a part missing, we imported the remained images into the dcm2nii program, and transformed them from DICOM to NII format for statistical analysis. In this study, one patient was excluded due to data quality problems. In total, 106 patients and 57 healthy participants were included. Resting-state fMRI images were preprocessed using the Data Processing and Analysis of Brain Imaging (DPABI) toolbox (http://rfmri.org/dpabi) (Yan *et al*., 2016). The steps were as follows: (i) discarding the first 10 volumes to stabilize the signal of the scanner and enable participants to adapt to the environment; (ii) slice timing correction for the rest 200 fMRI images; (iii) head motion correction (participants would be excluded if their maximal head motion exceeded 3 mm displacement or 3° of rotation); (iv) spatial normalization to standard Montreal Neurological Institute space and resampled to 3×3×3 mm^3^ resolution; (v) regression of nuisance covariates including the Friston-24 motion parameters, white matter signals, cerebrospinal fluid signals, and global signal; (vi) spatial smoothing using a Gaussian kernel with full-width at half-maximum of 8 mm; (vii) detrending; and (viii) temporal band-pass filtering at a frequency band of roughly 0.01–0.08 Hz.

### Dynamic ALFF analysis

The dALFF analysis was performed using the DynamicBC toolbox (Liao *et al*., 2014; Li *et al*., 2019a) and based on a sliding window method. Previous studies (Rong *et al*., 2018; Li *et al*., 2019a) proposed that the window width was an open but essential parameter in sliding-window-based resting-state dynamics computation. To avoid the introduction of spurious fluctuations, the minimum window length had to be larger than 1/*f*_min_, where *f*_min_ denotes the minimum frequency of time series (Leonardi and Van De Ville, 2015; Li *et al*., 2019b). Some scholars (Shakil *et al*., 2016; Savva *et al*., 2019) chose different window widths for verification, and found that the repeatability of retest analysis at 50TR was high. In addition, some scholars (Pang *et al*., 2018; Li *et al*., 2019b) believed that the window length of 50 TR was the best parameter to keep the balance between capturing the fast-changing dynamic relationship and obtaining reliable inter egional correlation estimation, which verified Leonardi's view. Hence, 50 TR (100 s) was selected as the sliding-window width and 1 TR (2 s) as step size to calculate the dALFF of each participant. The time series of each participant was divided into 151 windows and the ALFF map was computed within each window, generating a set of ALFF maps for each participant. Subsequently, the standard deviation of these maps was measured to evaluate the temporal variability of dALFF.

In addition, the ALFF results were also calculated as a comparison, and their data preprocessing procedure was the same as that for dALFF. The difference between ALFF and dALFF was that ALFF had only one window containing all time points.

### Statistical analysis

The dALFF variability value was averaged at each voxel across participants within CNP and HC groups to obtain a dALFF variability distribution. A two-sample *t*-test was performed to assess the group differences in dALFF variability between the two groups, with age and sex as covariates. Multiple comparisons were corrected using Gaussian random-field (GRF) method (*P* < 0.05).

To fulfil the requirements of a two sample *t*-test, the samples are independent, normal, and need to meet the homogeneity of variance (Ghurye, 1949); in this study, the two groups’ data were independent, and the normality could be ensured by *Z*transform. As the unbalanced sample size of the two groups could have influenced the heterogenetic variance, the test of the homogeneity of variances was taken, but the results failed to accept the homogeneity of variances. Therefore, the method of modifying the degrees of freedom was used to solve this problem (Ruxton, 2006). According to the statistical study by Ruxton (Ruxton, 2006), the adjusted *t*-statistic in the case of unequal variance is:

\begin{equation*}
{\mathrm{v\,\,}} = {\mathrm{\,\,}}\frac{{{{\left( {\frac{{s_1^2}}{{{n_1}}} + \frac{{s_2^2}}{{{n_2}}}} \right)}^2}}}{{\frac{{{{\left( {\frac{{s_1^2}}{{{n_1}}}} \right)}^2}}}{{{n_1} - 1}} + \frac{{{{\left( {\frac{{s_2^2}}{{{n_2}}}} \right)}^2}}}{{{n_2} - 1}}}}
\end{equation*}

### Correlation analysis

To further investigate the potential associations of abnormal dALFF variability with five pain factors in patients with CNP, namely average daily working hours at desk, average daily usage time of digital products, course of disease, average time of attack per day, and per week, the mean dALFF variability values of each ROI (see Table 2) were extracted to calculate the Pearson's correlation coefficient with pain-associated factors in patients with CNP. A statistically significant threshold of *P* < 0.05 was set for the correlation analysis.

**Table 2:** dALFF alterations between patients with CNP and HC.

Region name	Hemisphere	Peak coordinates	Peak *t* value
Middle Temporal Gyrus	Left	(−60 −51 12)	−3.13
Inferior Temporal Gyrus	Left	(−60 −54 −6)	−4.51
Angular Gyrus	Left	(−54 −66 27)	−3.61
Inferior Parietal Gyrus	Left	(−42 −63 54)	−3.24
Middle Occipital Gyrus	Left	(−33 −66 39)	−3.68

## Results

### dALFF variability results

**Figure 1:**
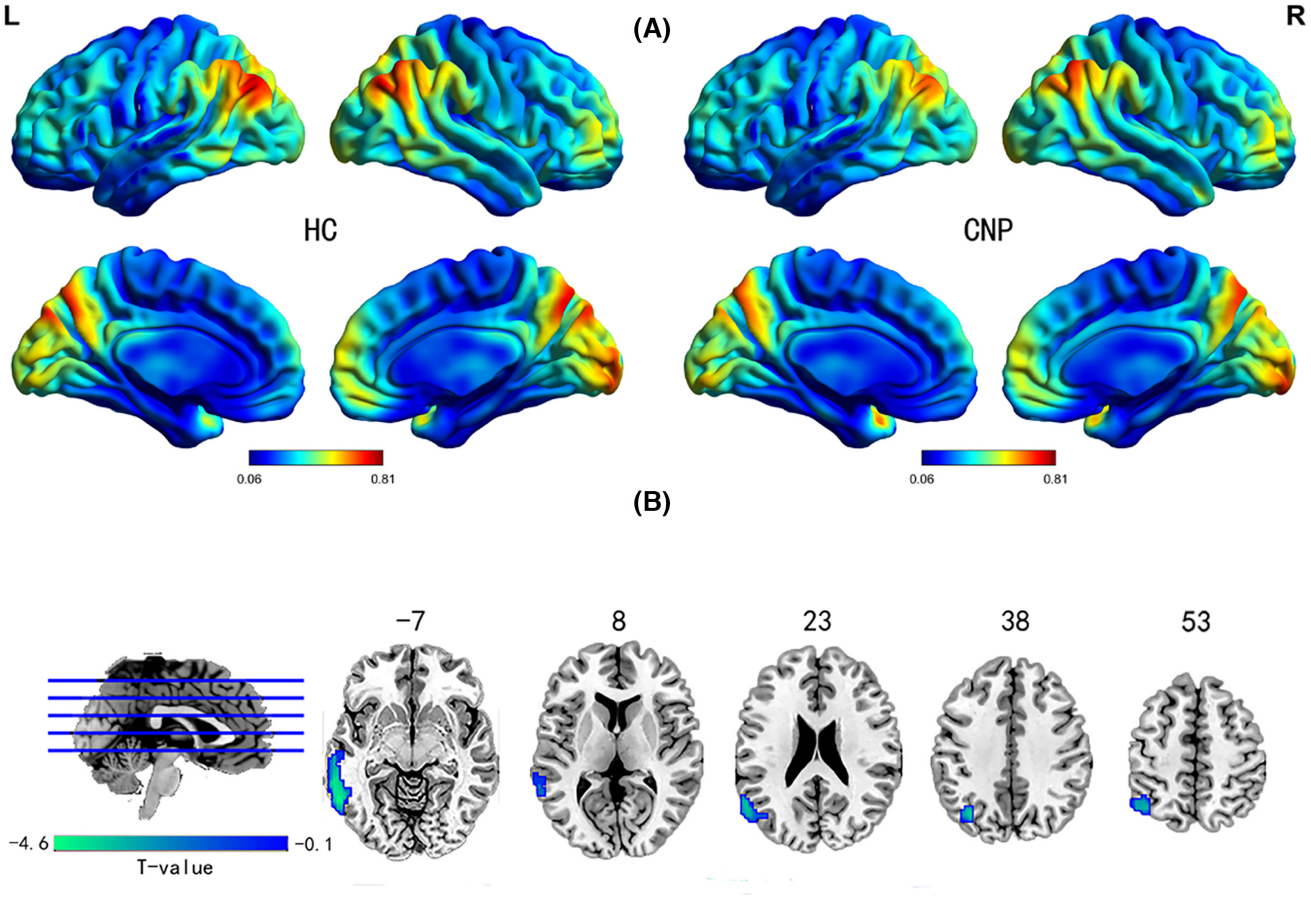
Group differences in dALFF variability between CNP and HC were identified using a two-sample *t*-test. The statistical significance level was set at *P* < 0.05, GRF corrected.

Similar spatial distribution of dALFF variability of patients with CNP and HCs is shown in Fig. 1a. The brain regions with high dALFF variability were mainly located in the temporal-parietal junction, the occipital cortex, and the prefrontal lobe. The group differences in dALFF variability between patients with CNP and HCs are shown in Fig. 1b. The results show that patients with CNP exhibited significantly decreased dALFF variability in the left inferior and middle temporal gyrus, the left angular gyrus, the left inferior parietal marginal angular gyrus, and the left middle occipital gyrus (*P* < 0.05, GRF corrected). The details of the peak information are listed in Table 2. Compared with HC, no brain region with increased dALFF variability in patients with CNP was found.

### Correlation results

**Figure 2:**
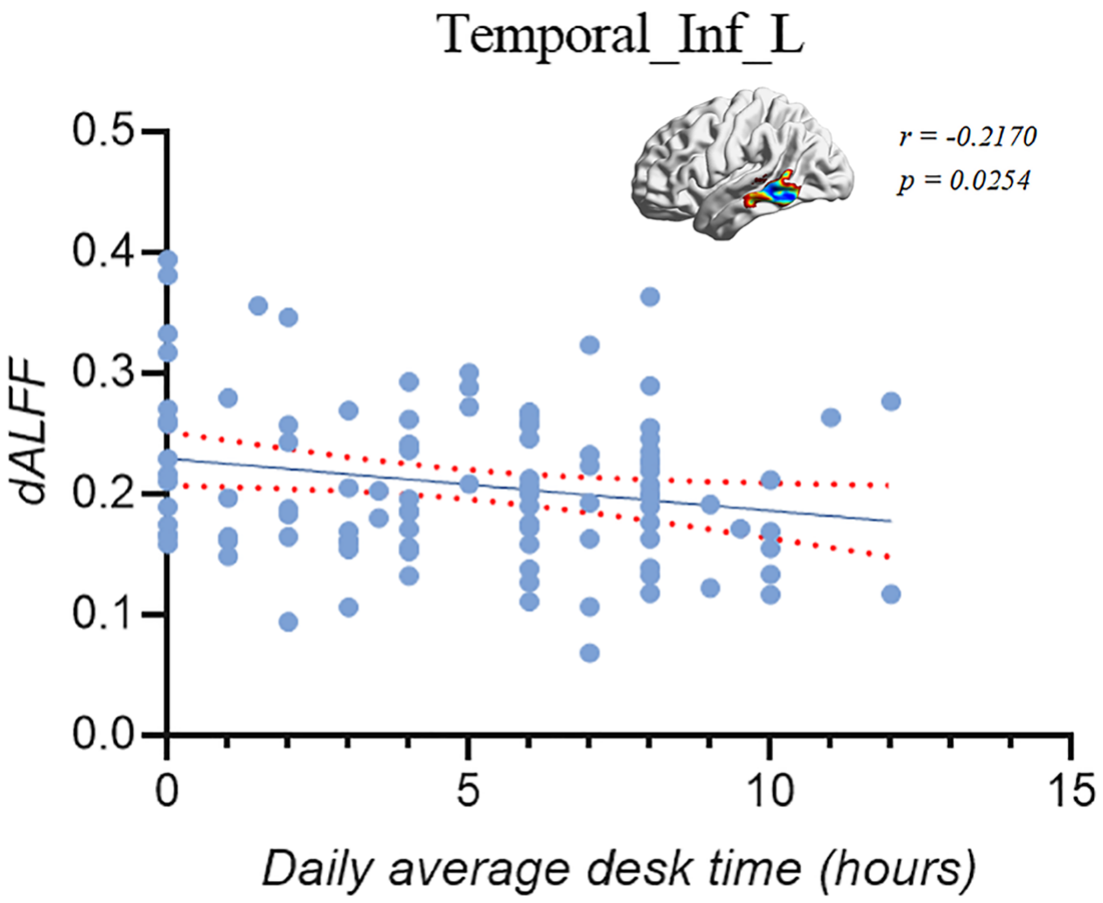
dALFF variability in the left inferior temporal gyrus (Temporal_Inf_L) was negatively correlated with the average daily working hours in CNP (*r* = −0.2170, *P* = 0.0254).

Fig. 2 shows that abnormal dALFF variability of the left inferior temporal gyrus was negatively correlated with the average daily working hours of patients with CNP (*r* = −0.2170, *P* = 0.0254). In addition, there was no other significant correlation.

### ALFF results

**Figure 3:**
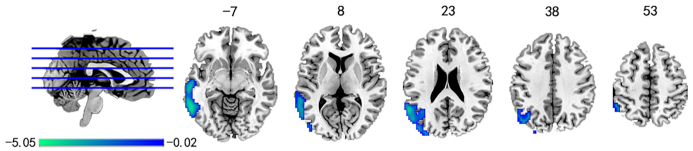
Group differences in ALFF values between CNP and HC by using a two-sample *t*-test. The statistical significance level was set at *P* < 0.05, GRF corrected.

The group differences in ALFF values between patients with CNP and HC are shown in Fig. 3. The results demonstrate that patients with CNP exhibited significantly decreased ALFF values in the left inferior and middle temporal gyrus, and the left angular gyrus (*P* < 0.05, GRF corrected). The details of the peak information are listed in Table 3. However, no significant correlation has been found between the abnormal ALFF values and any of the five pain factors in patients with CNP.

**Table 3:** ALFF alterations between patients with CNP and HC.

Region name	Hemisphere	Peak coordinates	Peak *t* value	Cluster size
Middle Temporal Gyrus	Left	(−63 −36 0)	−5.05	400
Inferior Temporal Gyrus	Left	(−60 −54 −6)	−4.77	39
Angular Gyrus	Left	(−57 −57 24)	−3.45	127

## Disscusion

It has been shown that ALFF could reflect the spontaneous functional activity of local brain regions in the resting state, indicating that this spontaneous neuronal activity has physiological significance and the information interaction between different brain regions produces its own rhythmic activity pattern (Biswal *et al*., 1995). In spontaneous neural activity (Deco and Corbetta, 2011), the characterization in the sustained changing of connections between brain regions could indicate a potential functional correlation (Tagliazucchi *et al*., 2012; Liu and Duyn, 2013). Therefore, the dynamic change of brain activity reflects the temporal variability of cognitive adaptability, which provides a theoretical basis for the research hypothesis of brain dynamic activity characteristics (Bassett *et al*., 2011; Fornito *et al*., 2012; Wang *et al*., 2016). Subsequently, the dALFF approach could contribute to revealing the different characteristics of the nervous systems associated with CNP, and further improve our understanding of the physiological mechanism of neck pain.

To our knowledge, the present study was the first time that alterations of the temporal variability of regional brain activity in patients with CNP were explored using a dynamic ALFF method. Dynamic ALFF is a measure of spontaneous activity of the brain in resting-state on a time scale. We found that in patients with CNP, dALFF variability significantly decreased in the left middle temporal gyrus, the inferior temporal gyrus, the angular gyrus, the inferior parietal marginal angular gyrus, and the middle occipital gyrus compared with HCs. In addition, the abnormal dALFF variability in the left inferior temporal gyrus was related to the factors leading to the severity of neck pain. These findings highlight the importance of considering dynamic local brain activity in patients with CNP.

It is worth noting that the parietal, temporal, and occipital lobes of the brain are involved in the processing of auditory and visual information as well as sensory and emotional information (Cogen *et al*., 1979; Karami *et al*., 2019; Hua *et al*., 2020). The middle/inferior temporal gyrus are located under the lateral fissure of the brain. They participate in the formation of the orbitofrontal temporal lobe limbic system, which mainly performs the self-control of social emotions and the management of emotional behaviors (Lim *et al*., 2014). Previous studies have found that patients who suffered from pain for a long time may also exhibit depressive symptoms (Lin *et al*., 2018; Maleki and Oscar-Berman, 2020; Song *et al*., 2020). Meanwhile, other imaging studies found that in a state of anxiety and depression, the relative cerebral blood flow of the temporal lobe region was decreased. However, the emotional response mechanism related to pain could be improved by increasing the relative cerebral blood flow in this region (Chen *et al*., 2004; Cooper *et al*., 2020). Both the angular gyrus and the inferior parietal marginal gyrus belong to the parietal lobe that located between the central sulcus and the parietooccipital sulcus. The parietal lobe participates in episodic memory retrieval, and only the left parietal lobe has this function (Phillips *et al*., 2009). Studies have shown that in painful diseases there are abnormal brain function activities in the parietal lobe, which is related to pain perception (Bolognini *et al*., 2013; Garza-Villarreal *et al*., 2015). Based on these results, regions showing temporal variability of abnormal brain activity not only regulate sensation and movement, but also play a role in cognitive function (Villar-Rodríguez *et al*., 2020) and are involved in pain related emotional regulation.

The decrease of ALFF values represented the same trend in spontaneous neuronal activity in the brain (Tan *et al*., 2018). Meanwhile, the decrease of dALFF values suggested that the local separation functions of these regions were impaired, and they were responsible for their specific behavioral functions (Siegel *et al*., 2016; Lee and Xue, 2017). Furthermore, the decrease of dALFF values also indicated that the flexibility of intrinsic spontaneous activity of brain was reduced. It represented less efficient information processing, and more erratic behavior and work ability with lower cognitive abilities (Kielar *et al*., 2016). Therefore, the overlapped abnormal brain regions in the ALFF and dALFF maps indicated that CNP might lead to slower processing of single-mode information and some uncontrollable behavior performance. There was a certain decline in work and cognitive ability, which implied that patients with CNP may have sensory and cognitive dysfunction at the same time (Chai *et al*., 2016; Duncan and Small, 2016; Kielar *et al*., 2016).

Moreover, there was a negative correlation between the dALFF values in the left inferior temporal gyrus and the daily average work time in the patient group. The longer the work time was, the lower the dALFF values in the left inferior temporal gyrus were. This suggests that the temporal variability of spontaneous activity of the inferior temporal gyrus decreased with the increase in working time, which might result in serious brain and nerve damage. Although this exploratory result was not corrected for multiple comparison (Cui *et al*., 2020), we speculated that the more severe the pain was, the lower the flexibility of brain activity in the inferior temporal gyrus might be. Subsequently, the adaptation of functional specificity was weakened and this might lead to the weakening of emotion regulation ability of pain, emotional disorder, anxiety, and depression (Lin *et al*., 2018; Maleki and Oscar-Berman, 2020; Song *et al*., 2020). Our findings laid a solid foundation to distinguish patients with CNP and HCs from the perspective of local brain activity, and provide new evidence to better understand the neural mechanism of CNP.

For contrast, the ALFF values were also calculated and compared between patients with CNP and HCs. ALFF values represent the static local spontaneous activity of a brain region over time, while variability of dALFF values represents the variance of the local spontaneous activity of the brain region over time. This paper found that the difference map of ALFF had a lot of overlap with that of dALFF, which showed clusters in the left middle temporal gyrus, the left inferior temporal gyrus, and the left angular gyrus. What is more, specific brain regions associated with altered dALFF were also found in patients with CNP, suggesting that dALFF represented complementary information compared with ALFF in patients with CNP.

Note that the voxel size of brain functional image was 3.75 × 3.75 × 5 mm. However, in the data preprocessing, the image was normalized to 3 × 3 × 3 mm. The resolution of the scanned image was larger than the normalized resolution value, and thus a partial volume effect might have appeared. All images were resampled with the resolution of the standard voxel after preprocessing, therefore the threshold value of head motion parameters was taken as one voxel being equal to 3 mm.

## Conclusions

To our knowledge, this was the first study that attempted to investigate the alterations of dynamic spontaneous activity in patients with CNP. The results demonstrated that patients with CNP had reduced temporal variability of ALFF in regions responsible for auditory, vision, memory, and emotion, which suggested the reduction of flexibility in these brain regions. In addition, abnormal ALFF variability in the left inferior temporal gyrus was associated with the risk factor of neck pain. This study explored the brain dysfunction of neck pain from the perspective of regional brain activity variability, which might give new insights into the neurophysiological mechanism of neck pain.
